# Experiences of people with Long Covid with a digital physiotherapy intervention: A qualitative study

**DOI:** 10.1111/hex.13993

**Published:** 2024-04-08

**Authors:** María‐José Estebanez‐Pérez, Rocío Martín‐Valero, Pablo Pastora‐Estebanez, José‐Manuel Pastora‐Bernal

**Affiliations:** ^1^ Department of Physiotherapy, Faculty of Health Science University of Malaga Málaga Spain; ^2^ Department of Physiotherapy Faculty of Health Science, University of Granada Melilla Spain; ^3^ Department of Economy, Faculty of Economic and Business Sciences University of Málaga Málaga Spain

**Keywords:** digital physiotherapy intervention, experience, Long Covid, perception, qualitative method

## Abstract

**Purpose:**

Long Covid syndrome is a multiorgan condition with multiple sequelae affecting quality of life, capacity to work and daily activities. The advantages that new technologies can offer are presented as an opportunity in the current healthcare framework.

**Objective:**

This research aimed to explore people with Long Covid's experiences with a digital physiotherapy practice intervention, during four weeks.

**Methods:**

Qualitative semistructured interviews were conducted by video call. Thirty‐two Long Covid participants were invited to join an in‐depth interview once the intervention was completed. Participants were queried on their intervention experiences and perceptions, as well as any lifestyle changes made, as a result of receiving digital physiotherapy practice. The interviews were transcribed and analysed using inductive qualitative content analysis.

**Results:**

In‐depth qualitative analysis has revealed four themes that reflect participants' perceptions of digital physiotherapy intervention. The helpfulness of the exercises, interaction with the physiotherapist, the domestic use of technology and the future of digital health practice were the topics highlighted by Long Covid participants. Some improvements have been suggested including video sounds and the need to introduce face‐to‐face sessions. Participants stated that interventions were helpful and superior to printed exercise sheets, mobile phone apps and usual care received. This intervention did not present major barriers, highlighting the importance of personalized care and continuity in the provision of health services.

**Conclusion:**

The digital physiotherapy practice is perceived by people with Long Covid as an appropriate method for the care of their health needs. Participants stated the need for this type of intervention in the public health system, where it would eliminate waiting lists, facilitate accessibility and improve existing care.

**Patient and Public Contribution:**

Participants contributed to the interpretation of the data acquired in the interview.

**Clinical Trial Registration:**

Trial registration NCT04742946.

## 1 INTRODUCTION

Long Covid is a recent syndrome characterized by the persistence of symtoms weeks or months after initial infection, or by the appearance of siymptoms after a period of time.^1^ Long Covid is estimated at 10%–20% of patients infected with SARS‐CoV‐2,^2^ where women aged around 40–45 years with not associated health problems have the hightes prevalence.^3^ Recently, up to 201 symptoms were presented in a study of Long Covid patients,^4^ with the most prevalent symptoms being fatigue, dyspnoea, myalgia, arthralgia, headache, cough, alteration to smell and taste and brain fog, as well as effects on mental health.^4^, ^5^

Digital physiotherapy practice is a term used to describe the remote delivery of rehabilitation services by physiotherapists using communication technologies,^6^ in consultations or home therapy.^7^ Digital physiotherapy practice has been expanding through the use of communication technologies, thanks to their rapid growth, and the decreasing costs of services. This has contributed to the integration of videoconferences, video games, virtual reality and other technological applications^8^ in healthcare.

During the Covid‐19 pandemic, the use of digital health interventions has increased considerably due to the difficulty of providing supervised face‐to‐face health care.^9^ Studies based on digital physiotherapy practice have published results of efficacy, validity, noninterference and significant benefits in several diseases,^10^ providing an alternative approach.

A recent study in patients with sequelae of Covid‐19 showed significant improvements based on individualized and supervised treatment.^11^ The World Health Organization (WHO) has issued self‐care recommendations for this new syndrome.^12^ The research team designed a 4‐week personalized and individualized digital physiotherapy programme for Long Covid patients. This intervention allowed participants to self‐manage the exercise programme, according to the subject's level of fatigue, before a training session.

Long Covid patients have described feeling physically and emotionally exhausted by the difficulties and barriers to accessing health services,^13^ some have even described not being taken seriously. It seems necessary to design new or adapt existing health services for people with Long Covid,^14^ and to assess the quality of care or, in other words, to identify the needs of this population.^15^

Qualitative methods are used to explore a deeper contextual understanding of participants' experiences and needs with different physiotherapy interventions,^16^ including digital physiotherapy practice.^17^ Interviews are a suitable method for obtaining valuable information about patients' experiences, through which researchers acquire in‐depth information.^18^ The research team found it appropriate to use a qualitative constructionist framework through semistructured interviews.

This study aimed to explore people with Long Covid experiences and perceptions with a customizable 4‐week digital physiotherapy intervention, with the intention of gaining insight into the challenges and possible barriers present during its realization.

## 2 MATERIALS AND METHODS

### 2.1 Study design

The present study was conducted according to the criteria for reporting qualitative research (COREQ).^19^ This study is embedded in larger research that also explored the functional capacity and adherence of a digital physiotherapy intervention in people with Long Covid.^20^ This research started at the end of a digital physiotherapy intervention during 4 weeks. The qualitative data was analysed using a thematic analysis approach by video call interviews. The research team chose semistructured qualitative interviews as the most appropriate type for participants, to talk in depth about their experiences of the intervention used.^21^

### 2.2 Participants

Participants were recruited by nonprobabilistic sampling, due to needs and characteristics of the subjects and the research, from various health centres in Andalusia by health collaborators (physiotherapists, nurses and family doctors from the reference centres). This study was carried out during the year 2022. The study includes adults (over the age of 18) with a diagnosis of Long Covid, ICD‐10 (U09) and ICD‐11 (RA02), as it has been designated by the International Classification of Diseases.^22^ Participants must have computer technology with an internet connection at home.

A total of 32 participants completed the 4‐week intervention and were invited to participate in an in‐depth interview, which was scheduled for the following week. Participation was voluntary. All participants (*n* = 32) agreed to be involved. Only 9.4% of the participants required hospitalization, and 6.3% of the participants required intensive care. Regarding gender, the majority of participants were women (71.9%). Age (range: 18–65 years) and symptomatology (fatigue, joint pain, shortness of breath, memory loss, headaches) were heterogeneous among participants at baseline. Regarding their professional situation, the highest percentages were on sick leave (50%) and working full time (31.25%) respectively. Participant characteristics are shown in Table 1

**Table 1:** Participants characteristics.

	Participants (*n* = 32)
Age (*M* ± SD)	45.938 ± 10.65
Gender woman, % (*n*)	71.9 (23)
Hospitalized, % (*n*)	9.4 (3)
ICU, % (*n*)	6.3 (2)
Sick leave, % (*n*)	50 (16)
Full‐time job, % (*n*)	31.25 (10)

Abbreviations: M, mean; SD, standard deviation.

### 2.3 Digital physiotherapy intervention

**Figure 1:**
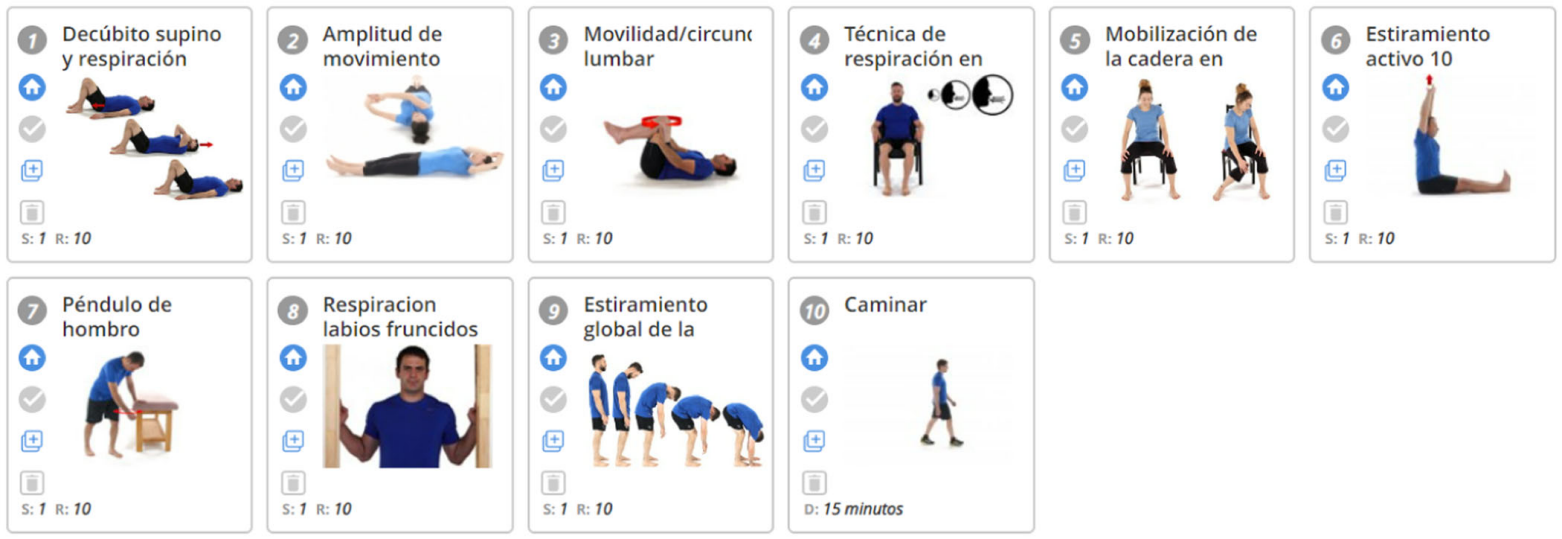
An example of the programme.

**Figure 2:**
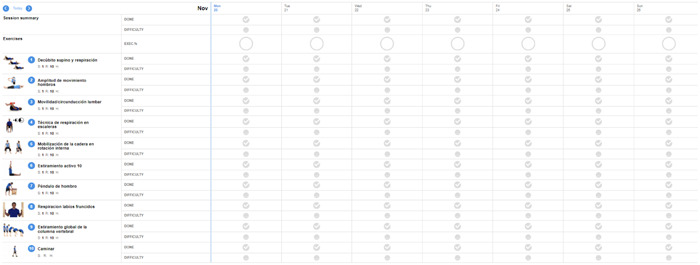
A follow‐up template.

Participants received a 4‐week personalized and individualized digital physiotherapy programme. The digital physiotherapy practice intervention includes the design of an individualized exercise programme, based on published clinical guidelines,^23^ reviewed and adapted weekly via video call and WhatsApp messaging. The research team selected the Physiotec software and mobile application, which allows the design of exercise programmes customized to the needs of the participants.^24^ An example of the programme and a follow‐up template are shown in Data S1 (Figures 1 and 2).

### 2.4 Data collection

The interview organized around a series of predetermined open‐ended questions, with other questions arising from the interviewer and interviewee's dialogue. Topics for the interviews included: motivation, personal experience, the digital physiotherapy practice, usefulness, changes, intention to use and future. The topic guide used in the research was added in Data S1.

Interviews were conducted in the participant's own home, after confirmation. Semistructured interviews were conducted by a researcher (P. P.‐E.) with experience in one‐to‐one interviews and without knowledge of the intervention performed. The interview duration ranged from 45 to 60 min at a single session. In the final phase, the interviewer briefly summarized the main themes discussed, and additional time was given for any comments or new ideas not previously exposed.^25^ Each interview was transcribed verbatim and anonymized for audit by the research team. Interviews were conducted online using the video‐conferencing application Google Meet.^26^ Inductive thematic analysis was used to analyse the data and identify the emergent themes.^27^

### 2.5 Data analysis

The relevant characteristics were coded taking into account the research question. Based on reflexive thematic analysis according to Braun and Clarke,^27^ we conducted an inductive analysis with the following steps: mapping the data set, data coding, initial theme generation, theme development and review, theme refining, defining, naming and writing up. The main researcher (M.‐J. E.‐P.) and the interviewer (P. P.‐E.) performed the first three steps.^28^ The researchers independently read all the transcripts multiple times to identify the codes. Inconsistencies in the coding were discussed until agreement was reached in weekly sessions. We conducted a team discussion to complete the rest of the steps. Different levels were collated (themes) based on similarities within the data and relationships between codes. The themes were rechecked, verifying that they worked with the entire data set, which allowed for inter‐subjective validation through comparisons and ensured the coherence and robustness of the analysis.^29^ Finally, the themes were named with the consensus of the research team. The emergent approach to data collection and analysis allowed us to determine when saturation had been reached. The data associated with the paper are available from the corresponding author on reasonable request.

## 3 RESULTS

Four core themes emerged from the thematic analysis concerning the outcomes perceived by participants after the digital physiotherapy intervention. The themes were labelled as *The helpfulness of the exercises*, *Interaction with the physiotherapist*, *Home use of technology* and *Future of the digital health practice*. Themes are described and contextualized below using representative, anonymized quotes (ID1, ID2, etc.).

### 3.1 Theme 1: The helpfulness of the exercises

Participants informed that the progression of the programme during the 4‐week intervention was especially meaningful and facilitated them to achieve the reported gains of their health. Overall, the digital physiotherapy programme helped them to achieve significant progress. Participants stated that having a structured and progressive intervention tailored to their needs was good and noted that the videos were helpful in reminding them of the technique.

Liked the progression from doing the exercises in bed to walking again. (ID10)

Finally, there was something or someone who could help us, trying to give a solution to all of this. (ID14)

Participants noted some benefits from exercise, with improvements in mobility (*n* = 9), pain (*n* = 6), flexibility (*n* = 3), mood/energy (*n* = 2), strength (*n* = 3), balance (*n* = 3), and pulmonary capacity (*n* = 6).

I've been feeling better and more encouraged. (ID5)

I had an active life, and after the Covid‐19 infection, I couldn't do anything. The exercises have helped me improve my feeling of fatigue, and finally, I can make my bed and sweep again. (ID7)

I have felt more stretched, I think I have recovered better from my second contagion, thanks to the exercise program. (ID12)

Three participants did not notice a substantial improvement.

Honestly, no, well it has given me moments of disconnection and relaxation. (ID23)

I have to admit that I have not experienced any improvement, but at least I have felt less lonely. (ID14)

Some participants (*n* = 14) compared with other interventions by stating that digital physiotherapy practice was superior to cell phone apps and printed exercise sheets. This was mainly due to the advantages of watching exercise videos and the awareness that the physiotherapist could monitor and progress the exercises by tailoring them to their needs.

Yes, because there is personalized attention, where the physiotherapist sees your needs and you can work with the exercises. In my medical visits they just gave me a piece of paper and that was it. (ID9)

My doctor sent me an exercise program. I started to do them and I did them with a lot of intensity and it annoyed me. (ID31)

### 3.2 Theme 2: Interaction with the physiotherapist

This theme refers to the fact that many of the participants felt abandoned or not taken seriously. This perceived need for support and health care occurred at the same time that they obtained online treatment. Participants reported feeling less lonely and more validated after using online support, thanks to the attitude and professionalism of the physiotherapist.

A significant percentage of participants (*n* = 22) expressed feeling hopeless and abandoned with their situation before starting the intervention. Most participants (*n* = 26) liked the WhatsApp and video call ‘contact’ with the physiotherapist, arguing that they could discuss their programme and solve any problems, so they felt it was not a generic website and motivated them to continue.

Finally, a physiotherapist gave a solution to all this that has been happening to me since I got Covid‐19. (ID12)

It is ideal to have a professional expert at your disposal. (ID32)

Participants reported that the physiotherapist made adaptations and changes to the programme, which allowed them to perform it without pain or overexertion. In addition, the physiotherapist provided tools and skills for the development of activities of daily living.

The physiotherapist helped me readjust the exercises, and showed me tricks to try to do something I didn't think I could do at first. (ID10)

The physiotherapist has helped me modify and adapt the execution, and I have started to be me again. (ID31)

At the beginning, the participants stated that they were less confident about how and when to perform the exercises, but with time and the help of the physiotherapist they have learned to adapt it to their routine and to overcome any obstacles or problems that may arise.

Over the weeks it has given me more confidence to do the exercise. And talking to my physiotherapist. (ID3)

I have felt very accompanied and cared for by the physiotherapist who has attended me, her empathy, warmth and availability has helped me get through the programme and improve. (ID12)

I give a 10 to the physiotherapist on duty. (ID13)

### 3.3 Theme 3: Home use of technology

The participants described the digital physiotherapy programme as easily accessible, safe and structured, which facilitated programme adherence. However, during the use of the home exercise programme, participants reported some incidences and potential barriers, due to technological deficiencies or the participants' difficulties in maintaining their concentration, or not knowing if they were performing certain exercises correctly.

Participants stated that they were generally satisfied with the digital physiotherapy intervention, confirming that the exercise training was useful and intuitive for them. All participants were happy to exercise at home, especially if they avoided new infections.

I could do the exercises whenever I want without leaving the house, I have already been infected twice. (ID22)

Participants were very comfortable with the type of intervention, but because of their loss of concentration and mental lapses (*n* = 5). They forgot the exercises they were doing in the programme and had to go back to the beginning.

I have had difficulties with the timing of the program. (ID8)

I found it difficult to hit done in the app, so it would register, because of my attention deficit and mental fog. (ID9)

In addition, participants were hesitant to perform breathing exercises despite having videos of them.

I had doubts about the breathing exercises. It would have helped to have sound in the videos. (ID3)

During the exercise I don't know if I have done the exercise properly. (ID5)

Regarding ‘digital physiotherapy practice’, participants stated that they enjoyed following the programme, they found they could do more than they thought they would and some added that they needed to do more treatment after the 4‐week intervention, which could also include face‐to‐face sessions.

I would add more time to the program, to keep moving forward. (ID9)

I think the digital physiotherapist service is ideal to intersperse with face‐to‐face treatment. (ID20)

### 3.4 Theme 4: Future of the digital health practice

Participants suggested improvements in the digital physiotherapy practice, with the intention of facilitating access and maximizing potential outcomes. All participants pointed out the need to include this type of technological tool in healthcare services.

Participants indicated improvements to be taken into account, such as audio, especially for breathing exercises, indicators that would make it easier to continue with the programme, or instant messages or sensors, about the execution of the programme.

Breathing exercises lack sound to understand them better. (ID3)

I would add an accompaniment during the exercises. (ID5)

The majority (*n* = 29) of participants stated that they planned to continue using their digital physiotherapy programme and specifically seven participants planned to return to activities they had abandoned when they became infected with Covid‐19.

Yes, because there is personalized attention, where the physiotherapist sees your needs and you can work with the exercises. Nowadays there is nothing like that, not that I know of. (ID11)

Participants stated the need for this type of intervention in the public health system, where it would eliminate waiting lists, facilitate accessibility, and improve existing care.

I would make it compulsory. (ID2)

It should be used now and so many waiting lists would be avoided. (ID14)

## 4 DISCUSSION

This qualitative study increases the knowledge available on people with Long Covid's experiences and perceptions with a digital physiotherapy intervention, addressing the main objective of the research. The information obtained reveals possible barriers or difficulties to work on, to favour its implementation.

The first theme, ‘*The helpfulness of the exercises*, reports how Long Covid participants had a positive experience of a 4‐week digital physiotherapy programme that allowed them to participate in their own recovery, as recommended by the World Health Organization,^12^ with the support of the physiotherapist through video calls and messaging. In this regard, self‐management of health has proven to be very important in the management of different conditions,^30^ and involving patients in their treatments is becoming more important nowadays, and digital physiotherapy intervention seems like an interesting way to achieve this.^31^

Patient feedback and end‐user perspectives provide important information regarding this new delivery intervention model, which may have a substantial impact on future uptake.^32^ In this research, the qualitative phase showed that all participants found the use of the programme useful, thanks to its benefits and progression. At this point, we have to take into account that three participants did not feel any improvement in their symptomatology, although they felt accompanied (*n* = 2) or more relaxed and entertaining (*n* = 1). In a systematic review including 45 studies with 9751 participants with Long Covid, 22.1% reported anxiety and 14.9% reported depression, among the most prevalent symptoms.^33^ Other systematic reviews found that therapeutic exercise is a means of reducing symptoms, such as anxiety and depression.^34^ Therapeutic exercise was included in our personalized programme.

In general, participants pointed out that the programme's adaptation throughout the 4 weeks of intervention was beneficial; they felt more comfortable with the intervention and confident. Furthermore, individualization was crucial for the implementation of this digital physiotherapy intervention, as has been previously argued,^35^ by the particularities of people with Long Covid. As presented in a previous study, physiotherapists should be responsive at all times to the health needs of their patients, promoting physical and mental well‐being.^36^ In this study, technology has made it easier for the physiotherapist to be very present during the development of the intervention.

The daily follow‐up of this intervention contributed to motivating participants. Motivation is also an essential factor in getting patients engaged in their health management.^37^ We found that motivation during the digital physiotherapy programme was facilitated by knowledge of the intervention, and continuous communication and support by the healthcare provider.

Regarding ‘the interaction with the physiotherapist’, the attitude and professionality of the healthcare provider managed to help the participants feel more accompanied and valued after the intervention. They appreciated the personalized attention in such uncertain and difficult times. Communication with the physiotherapist is a crucial aspect of health treatments.^38^ and may have been considered as difficult or uncomfortable, through technology devices. However, that has not been the case in this study. These statements are supported by data obtained in interviews, where the work of the physiotherapist was highly valued. Previous studies showed similar results with group digital intervention,^39^ where the instructors added a sense of fun to the group, which made it more enjoyable.

Participants have been suffering from persistent symptoms from half a year to one and a half years, without having received physiotherapy treatment before the intervention. Digital physiotherapy made it possible for participants on sick leave or working full time to take part in the exercise programme, highlighting the accessibility of the intervention. In general, participants reported that the intervention allowed them to carry out the exercise programme at home according to their preferences, rating it as very comfortable. This may be the reason for such positive results.

In this point, our research possible improvements were indicated during the ‘use of the technology at home’. A minority number of participants expressed doubts about the correct performance of certain breathing exercises, despite considering them generally very intuitive and easy to perform. The use of sound in the videos or sensors could clarify existing questions at that moment.

Healthcare software often appears to be underutilized. Subjects may feel a lack of confidence with technology if problems arise during its proper use, such as technological difficulties or problems of accessibility. Patients are generally more willing to use digital services, if they understand the benefits they bring to their health. Therefore, many of these problems could be solved with more support and knowledge from healthcare providers,^40^ based on the participant's self‐efficacy. The results of the interview analysis using a qualitative approach indicate that the digital physiotherapy programme has been perceived as fundamentally different from other home‐based exercise programmes, as printed exercise sheets or mobile phone apps.^41^ Participants state that the support of the physiotherapist makes a difference.

Regarding the theme ‘The future of digital health practice’, our research reveals how participants have strongly recommended, that this type of intervention may be included in public health services. At a time when healthcare is immersed in a phase of transformation, digital health is gaining ground, presenting itself as a solution to improve and expand healthcare services, setting the patient at the centre of care.^42^ The healthcare system has an important role to play in increasing access to digital resources, and thereby ensuring a digital service, that is tailored to the individual needs of patients.^43^ This theme is aligned with previous studies that suggested quality principles for an expanded Covid service, which included ensuring access to care, reducing the burden of disease, clinical accountability and continuity of care.^13^

Finally, the role of digital physiotherapy intervention can provide participants with the knowledge and self‐efficacy to manage health, through an individualized exercise programme,^41^ and may include face‐to‐face sessions, as has been suggested.

### 4.1 Limitations and strengths of the study

This study provides insights into the views and experiences of an intervention for a specific condition and thus does not claim to be generalizable,^16^ in common with most qualitative studies, which should be taken into account.

Patients without access to the technology may find it difficult to participate in this intervention, which may lead to selection bias. Moreover, technological difficulties (disconnection, device failures) that may arise during the course of the intervention must be considered.

Regarding strengths, the interviewer had no prior knowledge of the intervention performed, avoiding possible biases. In addition, the speed of the implementation process was guaranteed, because of the participant's use of their own technological devices. Our intervention generates few obstacles as it is available on any device that allows internet connection, allowing access from any location and different devices.^44^ The Internet and Information and Communication Technologies allow the patient to be more actively involved in their treatment, thus helping to create a communicative environment between the patient and the physiotherapist, which can be considered as a strength.^45^ Participants reported that they did not experience any adverse effects, such as increased fatigue, pain, and so forth, during the digital physiotherapy intervention, so we may consider our intervention to be feasible and safe. This statement is in line with previous digital health research with different study populations.^46^ Moreover, digital physiotherapy practice has been suggested as a strategy during pandemic^47^ and its sequelae.

## 5 CONCLUSIONS

The digital physiotherapy practice is perceived by people with Long Covid as an appropriate method for the care of their health needs, with no major barriers to its use, and highlights the importance of personalized care and continuity in the provision of health services. Participants stated the need for this type of intervention in the public health system. However, further qualitative and quantitative research with larger samples and control groups is needed to draw extrapolable conclusions.

## AUTHOR CONTRIBUTIONS

**María‐José Estebanez‐Pérez**: Conceptualization; investigation; methodology; writing—original draft; writing—review and editing; formal analysis. **Rocío Martín‐Valero**: Supervision; writing—review and editing; investigation. **Pablo Pastora‐Estebanez**: Formal analysis; writing—review and editing; investigation; methodology. **José‐Manuel Pastora‐Bernal**: Writing—review and editing; investigation; supervision; conceptualization.

## CONFLICT OF INTEREST STATEMENT

The authors declare no conflict of interest.

## ETHICS STATEMENT

The fundamental ethical precepts according to the Declaration and Law of Helsinki 14/2007 of July 3 on Biomedical Research have been respected, guaranteeing the protection and confidentiality of data. Only researchers had access to the data. This trial has the approval of the Andalusia Ethics Committee with HIP version 281020. The researcher declares that they follow the protocols of his work centre regarding the publication of data in accordance with the provisions of Organic Law 15/1999, December 13, on the Protection of Personal Data (LOPD), and that the data were incorporated into a file for the purpose of carrying out this research project. Participating subjects were informed of the possibility of exercising their rights of access, rectification, cancellation and opposition of their data at the e‐mail address provided by the principal investigator. An informed consent was signed by all the subjects participating in the study, having previously received sufficient information about the objectives and procedure of the study. They were also informed of the possibility of revoking the consent given at any time, without having to justify their decision and without prejudice. The research team requested the informed consent of the subjects referred to in the research project. The consent form is added in Data S2.

## Supporting information

Supporting information.

Supplementary Information

## Data Availability

The data that support the findings of this study are available from the corresponding author upon reasonable request.
