# Supplementary material for: Experiences of people with Long Covid with a digital physiotherapy intervention: A qualitative study
Source: Health Expect. 2024 Apr 8;27(2):e13993. doi: 10.1111/hex.13993 (PMC11002316; doi:10.1111/hex.13993)
Supplement: Supplementary file 1 — Supporting information. [file HEX-27-e13993-s001.docx]

Interview

**Opening preamble for each interview:** Thank you for agreeing to take part in an interview today with regard to the research study on the digital physiotherapy practice in long covid patients. We will both need to sign and date the consent form before we proceed with the interview.

Motivation

How did you hear about the study?

What interested you about the project to make you want to take part?

Intervention

What was your experience with using the telephone therapy programme?

What was easy and what was difficult?

In the four weeks of the digital physiotherapy practice, what did you like and dislike, and why?

Did the physiotherapist help you to clarify any doubts? If so, how?

If you had to choose one word to define the physiotherapy approach to your condition through a digital physiotherapy practice, what would it be?

Digital physiotherapy practice

What is your skill level with computers, smartphones, etc.?

Were there any aspects where communication was difficult because of the use of the Digital physiotherapy practice?

Was there anything that you felt uncomfortable saying or doing over the Digital physiotherapy practice?

Which tool did you find most useful and which less useful, and why?

Usefulness

How did the attitudes of your family and friends influence your use of the digital physiotherapy practice?

When did you use it (in which time slot) and how do you remember using it? Did you use the system alone, together with family members?

How did you manage the obstacles?

Changes

Did you notice any improvements or changes with the use of the Digital physiotherapy practice over time, and did you notice any changes in your motivation and attitude towards using the programme over time?

Has the programme been able to help you improve your ability to carry out your daily household/work/sport activities? How?

Intention to use

Would you consider using this programme again, and for what reason?

Would you recommend participating in the post-Covid-19 tele-physiotherapy project to an acquaintance or friend who could participate? why?

Future

How would you improve the Digital physiotherapy practice you have received for 4 weeks?

What would you remove from the Digital physiotherapy practice?

What would you add about the Digital physiotherapy practice?

Do you think that Digital physiotherapy practice could be a useful tool within the social security system, and if so, why?

**Close of interview:** Thank you very much for participating in this interview.
