# Supplementary material for: Experiences of people with Long Covid with a digital physiotherapy intervention: A qualitative study
Source: Health Expect. 2024 Apr 8;27(2):e13993. doi: 10.1111/hex.13993 (PMC11002316; doi:10.1111/hex.13993)
Supplement: Supplementary file 2 — Supplementary Information [file HEX-27-e13993-s002.docx]

Informed consent - Written consent of the patient

COVID-19 can cause important sequelae in the patient after overcoming the virus, such as: fatigue, joint pain, headaches, sleep disorders and respiratory problems, among other symptoms. Physiotherapy has been shown to play a fundamental role in the recovery of functions and quality of life and its effectiveness in patients who have been infected by this virus is under continuous study.

Digital physiotherapy practice is a term used to describe the provision of rehabilitation services at a distance, using communication technologies, such as mobiles, tablets and computers, which can better meet the needs of the patient, in terms of ease of access and elimination of travel and in the case of COVID-19 limiting its potential transmission by avoiding direct person-to-person contact. Digital physiotherapy practice has increased significantly during periods of confinement and is presented as an intervention opportunity for patients.

WHAT DOES THIS RESEARCH PROJECT CONSIST OF?

We want to test the effectiveness of Digital physiotherapy practice in Long COVID-19 patients for the improvement of their functional capacities. We also investigated the level of adherence to treatment.

Patients participating in the research are assessed by the research team to identify their particular health needs. For 4 weeks, they will receive personalised physiotherapy sessions in digital format using technological tools via their mobile phone, computer or tablet. The researchers will inform them of the recommendations to be followed during the evolution of the sessions and the treatment to be carried out after the treatment period.

The research project has been approved by the Research Ethics Committee of Andalucía. The interventions to be carried out have the maximum guarantees of good professional practice, safety and data protection. Your case will be closely monitored by the researchers, who include professionals from the Universities of Malaga and Granada with more than 25 years of professional and research experience.

WHAT DO YOU NEED TO PARTICIPATE?

- Be of legal age

- Diagnosis: Long COVID-19

- Have a mobile phone, tablet or computer at home and an internet connection.

- Complete and sign the informed consent document attached below.

INFORMED CONSENT - WRITTEN CONSENT OF THE PATIENT

I (Name and surname): ...................................................................................................

1. I declare that I have read the Patient Information Sheet that accompanies this consent.

2. I was able to ask questions about the study. All questions were answered to my satisfaction.

3. I have spoken to the reporting health professional: …………………………………………………

4. I understand that my participation is voluntary and I am free to participate or not in the study.

5. I have been informed that all data obtained in this study will be confidential and will be treated in accordance with the provisions of Organic Law 3/2018 of 5 December.

6. I understand that I can withdraw from the study:

- Whenever you want

- Without having to explain

- Without impacting on my medical care

I freely give my agreement to participate in the project entitled " Digital physiotherapy practice in Patients with Long COVID-19 ".

I DO

I DO NOT GIVE

Signature of the patient Signature of the informing health professional

First and last name: ........................... First and last name: ………………………………

Date: ..................................................

Contact telephone: .............................
